# Plasmids pick a bacterial partner before committing to conjugation

**DOI:** 10.1093/nar/gkad678

**Published:** 2023-08-18

**Authors:** Gad Frankel, Sophia David, Wen Wen Low, Chloe Seddon, Joshua L C Wong, Konstantinos Beis

**Affiliations:** Department of Life Sciences, Imperial College, London, UK; Centre for Genomic Pathogen Surveillance, Big Data Institute, Li Ka Shing Centre for Health Information and Discovery, University of Oxford, Oxford, UK; Department of Life Sciences, Imperial College, London, UK; Department of Life Sciences, Imperial College, London, UK; Rutherford Appleton Laboratory, Research Complex at Harwell, Didcot, Oxfordshire OX11 0FA, UK; Department of Life Sciences, Imperial College, London, UK; Department of Life Sciences, Imperial College, London, UK; Rutherford Appleton Laboratory, Research Complex at Harwell, Didcot, Oxfordshire OX11 0FA, UK

## Abstract

Bacterial conjugation was first described by Lederberg and Tatum in the 1940s following the discovery of the F plasmid. During conjugation a plasmid is transferred unidirectionally from one bacterium (the donor) to another (the recipient), in a contact-dependent manner. Conjugation has been regarded as a promiscuous mechanism of DNA transfer, with host range determined by the recipient downstream of plasmid transfer. However, recent data have shown that F-like plasmids, akin to tailed *Caudovirales* bacteriophages, can pick their host bacteria prior to transfer by expressing one of at least four structurally distinct isoforms of the outer membrane protein TraN, which has evolved to function as a highly sensitive sensor on the donor cell surface. The TraN sensor appears to pick bacterial hosts by binding compatible outer membrane proteins in the recipient. The TraN variants can be divided into specialist and generalist sensors, conferring narrow and broad plasmid host range, respectively. In this review we discuss recent advances in our understanding of the function of the TraN sensor at the donor-recipient interface, used by F-like plasmids to select bacterial hosts within polymicrobial communities prior to DNA transfer.

## Graphical Abstract

**Graphical Abstract:**
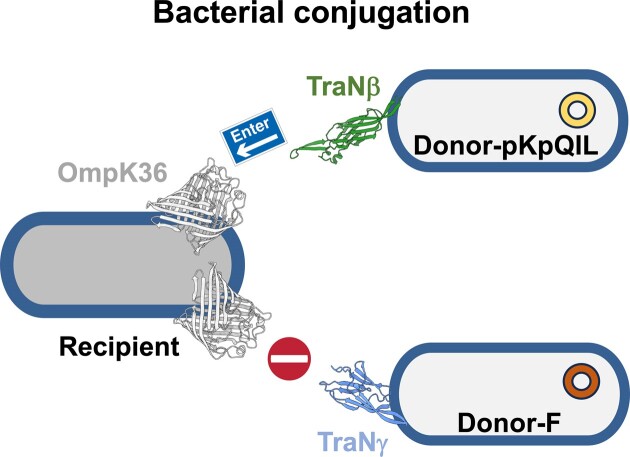
Mating pair stabilization and conjugation species specificity is mediated by specific pairing of a TraN sensor in the donor with an outer membrane protein in the recipient.

## INTRODUCTION

Conjugation, like transduction and transformation, is central to bacterial evolution, as it facilitates the acquisition and dissemination of virulence and antimicrobial resistance (AMR) genes (1). Conjugation is known to take place in a broad range of environments, including soil, the surface of plants and medical devices as well as in the lumen of the gut, which is considered a hot spot of gene exchange in bacteria of significant clinical importance (2,3). Since the discovery of conjugation almost 80 years ago (4), many functional studies have been performed using donor and recipient *Escherichia coli* with F or F-like (IncF) plasmids, which are predominantly isolated from *Enterobacteriaceae* (5). pMAR7, encoding the bundle forming pilus (BFP) in typical enteropathogenic *E. coli* (EPEC) and pSLT, encoding type III secretion system (T3SS) effectors in *Salmonella enterica*, are key F-like virulence plasmids (6,7). R100, first identified in an isolate of *Shigella flexneri* in 1956, encoding multiple resistance genes, and pKpQIL, found in current high risk *Klebsiella pneumoniae* sequence types (e.g. ST258/ST512), encoding the KPC carbapenemase, are classical and contemporary resistance F-like plasmids, respectively (8,9).

It has long been known that tailed *Caudovirales* bacteriophages (phage) use receptor-binding proteins (RBPs) at the distal end of their tail structures to bind specific bacterial surface polysaccharides (e.g. lipopolysaccharide (LPS) or capsule) and/or proteinacious receptor/s, prior to injection of their DNA. For example, the *Siphoviridae* phage λ binds LamB, while the *Myoviridae* phages T2 and T4 bind OmpA/FadL and OmpC, respectively (reviewed in (10)). Recent studies have shown that the *K. pneumoniae Siphoviridae* phages NPat and BMac bind OmpK36 (OmpC homologue) as well as the K2 capsule (11), while the *Serratia* sp. ATCC39006 LC53 phage (T4-like) seems to bind OmpW (12). These interactions determine transduction host specificity and range. In contrast, conjugation of IncF plasmids has been regarded as a promiscuous mechanism of DNA transfer, with host range being determined wholly by the recipient, downstream of plasmid transfer, via replication associated factors (e.g. incompatibility groups) (13), and restriction modification and CRISPR Cas systems (14).

**Figure 1.**
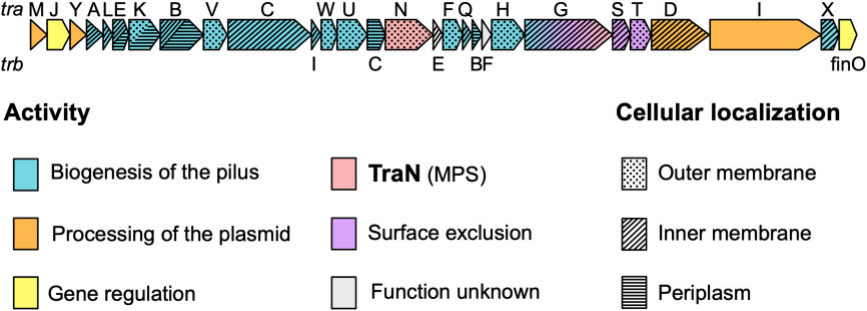
Genetic arrangement, function and subcellular localization of each gene products encoded by the F plasmid *tra* operon.

The transfer (*tra*) genes in conjugative IncF plasmids comprise a contiguous operon of approximately 40 kb (15) (Figure 1). The conjugation process itself can be divided into three phases. The first phase occurs exclusively within the cytosol of the donor. This includes assembly of the conjugative transfer machinery, related to type IV secretion systems (T4SS) (16), which facilitates assembly of the sex pilus (17). The second phase involves both the donor and recipient, starting with pilus-mediated mating pair formation (MPF) followed by TraN-mediated mating pair stabilization (MPS) (18). Plasmid transfer and the expression of plasmid-encoded genes in the recipient initiate the third phase of conjugation, which renders the recipient refractory to a second wave of conjugation by the same plasmid. TraT (localized to the outer membrane (OM)) and TraS (found in the inner membrane) participate in this process via mechanisms known as surface and entry exclusion, respectively (19,20). Surface and entry exclusion protect cells from lethal zygosis, where recipients are killed due to membrane damage when they partake in excessive conjugation activity (21).

## THE DONOR - RECIPIENT INTERFACE

### Mating pair formation

The sex pilus is an indispensable constituent of F and F-like plasmid conjugation; it is built as a thin flexible filament composed of polymerized pilin subunits encoded by *traA* (22,23). The F plasmid 121 amino acid TraA pro-pilin is cleaved into a 70 amino acid mature pilin subunit by TraQ and TraX (24). These pilin subunits accumulate in the inner membrane before they are assembled by 11 *tra* gene products. TraL, E, K, C and G participate in formation of the pilus tip (25). TraB, V, W, F and H are needed for extension of the pilus, TrbC is necessary for pilus biogenesis, although its precise function is unknown, and TraP stabilizes the extended filament. The TraI relaxase nicks the plasmid at the *oriT* site leading to formation of a TraI-ssDNA complex, which is recruited to the T4SS by the coupling protein TraD that initiates DNA transfer (19,20); TraU also plays a role in DNA transfer, while TrbI plays a role in pilus retraction (26–31).

The structures of the sex pili encoded by the F and F-like plasmids pED208 and pKpQIL have been determined by single-particle cryo-EM (32,33). This revealed that the pilin subunits form helical assemblies with phospholipid molecules at a stochiometric ratio of 1:1 (32,33). The lumen of the sex pilus is ∼25 Å in diameter, the external diameter is ∼85 Å and the average length is 20 μm (32,34). While the structure of the pilus has been determined, our understanding of its role in conjugation remains incomplete. It is broadly recognised that the pili are important for the initial contacts between the donor and recipient during MPF (35). However, the molecular basis of MPF remains unknown as the pilus receptor on the recipient has not yet been identified, partially because the composition of the pilus tip remains undefined. Using live-cell fluorescence microscopy, Clarke *et al.* demonstrated that the pilus is a highly dynamic structure and that pilus-mediated interaction between two Hfr bacterial cells triggers its retraction leading to the formation of cell–cell contacts (36). Babic *et al.* provided evidence that plasmid DNA can be transferred from a donor to a distant recipient (37). Recently, Goldlust *et al.* have shown that distant plasmid transfer occurs through the center of the pilus, answering a long-standing key question in conjugation biology (doi: https://doi.org/10.1101/2023.06.21.545889).

### Mating pair stabilization

A key publication by Achtman and colleagues in 1978 had shown that following F pilus retraction, conjugating cells form mating aggregates that are resistant to disruption by shear forces (38), which bacteria may encounter in different niches, such as peristalsis in the gut (39). Close inspection of conjugating bacteria revealed that they form tight ‘mating junctions’, characterised by intimate wall-to-wall contact through a process later termed mating pair stabilization (MPS) (40). Initially, MPS was hypothesized to mediate an interaction between the tip of the pilus in the donor and a recipient receptor. However, it was later found that mutations in *traN* and *traG* affected the formation of mating aggregates without affecting the pilus (41), suggesting that intimate wall-to-wall contact is distinct from pilus-mediated MPF.

TraG is a multifunctional inner membrane protein (42). While its N-terminus plays a role in pilus assembly, the C-terminus is required for MPS; however, the mechanism by which TraG contributes to MPS remains elusive. TraN encoded by the F plasmid is a 602 amino acids (aa) outer membrane protein (OMP) that consists of 22 cysteine residues, of which six are important for optimal plasmid transfer (43,44). TraN encoded by the F-like plasmids pED208 and pOX38 was recently reported to contribute quantitatively to pilus production, conjugation efficiency and pilus extension/retraction dynamics (45).

**Figure 2.**
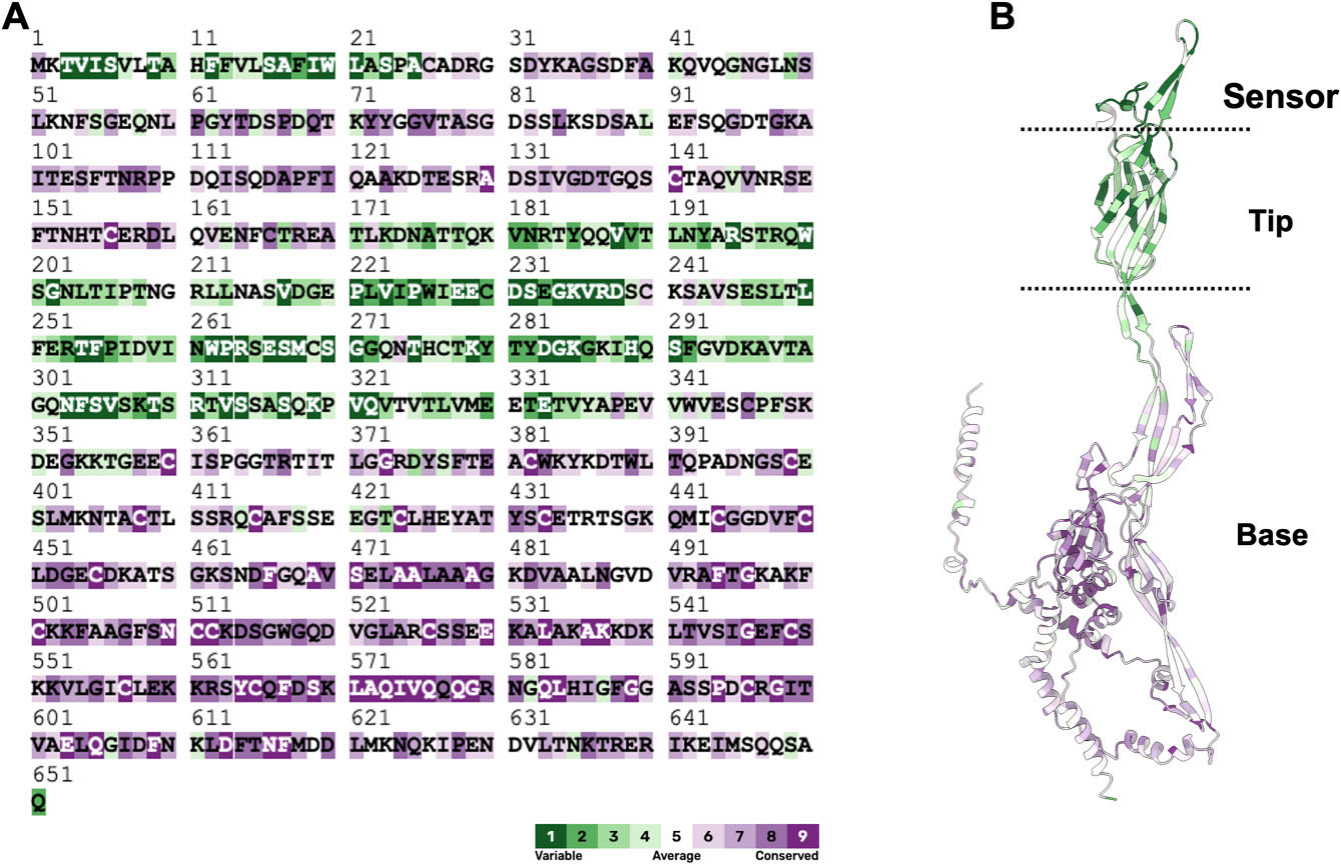
Conservation analysis of TraN. Sequence conservation of TraNs mapped onto the TraN encoded by the pKpQIL, as calculated by Consurf (61,62) (**A**) and the AlphaFold model (**B**). The conservation increases from green to purple. TraN is divided into three functional regions: the base, which anchors the protein to the outer membrane, the scaffold tip, and a distal sensor. The base shows the highest degree of sequence conservation whereas the tip and sensor the least.

While TraN shares little sequence identity with other known OMPs or adhesins, Klimke *et al.* determined its membrane topology and revealed the existence of three extracellular loops, which were predicted to be involved in receptor recognition (44). These loops correspond to a region spanning around 200 amino acids, which shares low sequence similarity between TraN homologues, while the N- and C-terminal domains are highly conserved amongst F-like plasmids. (Figure 2A). Importantly, the TraN homologues of a similar size (∼600 aa) are only found in IncF plasmids. In other plasmids MPS is mediated by different mechanisms.

MPS in the *Salmonella* Typhimurium plasmid R64 (IncI) is mediated by a thin flexible type IV pilus (T4P) encoded by the *pil* locus (located upstream of the *tra* operon). Donor - recipient interactions are mediated by binding of PilV, located at the tip of the T4P, to LPS. Inversion within the shufflon can form seven different PilV adhesins that bind specific LPS moieties on different recipients (46,47). In the *Enterococcus faecalis* pheromone-inducible conjugative plasmid pCF10, mating aggregates are formed by interactions between the plasmid-encoded aggregation substance protein PrgB on the donor and lipoteichoic acid on the receipt (48). In contrast, no specific recipient factors have been identified for conjugation of the broad host range plasmids R388 (IncW) and RP4 (IncP) (49,50).

### TraN sensors in the donor cooperate with distinct OMPs in the recipient

Studies intending to discover the sex pilus receptor in the recipient identified mutations in LPS biosynthetic genes, particularly the LPS core, and *ompA*, encoding the OMP OmpA, as negatively affecting conjugative uptake of the F plasmid (51). Three classes of *ompA* mutations were identified (52): mutants not expressing OmpA, mutants expressing lower levels of OmpA and mutants encoding missense mutations including a G154D substitution (53). Moreover, mutations in *ompA* were found to specifically affect transfer of the F plasmid but not the related F-like plasmid R100-1 (54). Building on this specificity, seminal work from the lab of Laura Frost in the 1990s found that OmpA was not the receptor for the sex pilus, as substitution of *traA* on the F plasmid with *traA* from the R100-1 plasmid did not abrogate the effect of OmpA mutations (55). Instead, the *ompA* mutations affected MPS, as dependency was associated with TraN, specifically the highly variable region of the protein (55).

The cooperation between the F plasmid TraN and OmpA in *E. coli* conjugation was recently confirmed, however, the F plasmid TraN did not recognise OmpA in a *K. pneumoniae* recipient (56). This, together with the finding that substitution of the F plasmid *traN* with *traN* of the R100-1 plasmid bypasses the dependency on OmpA (55), suggested that TraN–OMP interactions mediate conjugation specificity. These results also suggest that the recipient is not merely a bystander but instead participates in MPS.

Analysis of TraN sequences from 824 putative conjugative IncF-like plasmids in *Enterobacteriaceae* isolates revealed that 32%, 20% and 22% of plasmids encoded a TraN sharing ≥90% amino acid similarity to TraN of the pKpQIL, R100-1 and F plasmids, respectively (56). Analysing the remaining 215 plasmids led to the identification of four other TraN variants, one of which was found solely in *Salmonella enterica* serovars and specifically aligned to TraN from the virulence plasmid pSLT. The other three minor variants, labelled MV1-3, were not associated with well-known plasmids (56). Of note, while the 22 Cys residues of the F plasmid TraN are conserved across the family, the Cys content of TraN in different F-like plasmids can be >22 (56).

In the absence of an experimentally determined structure, the different TraNs were subjected to AlphaFold structural prediction (57). The TraN structure is composed of two domains: the base and an extended tip, which is composed of the highly variable sequence of the protein (Figure 2A and B). The 22 conserved Cys residues are found within the base domain and predicted to be paired via disulphide bonds, which could stabilise the structure (56).

**Figure 3.**
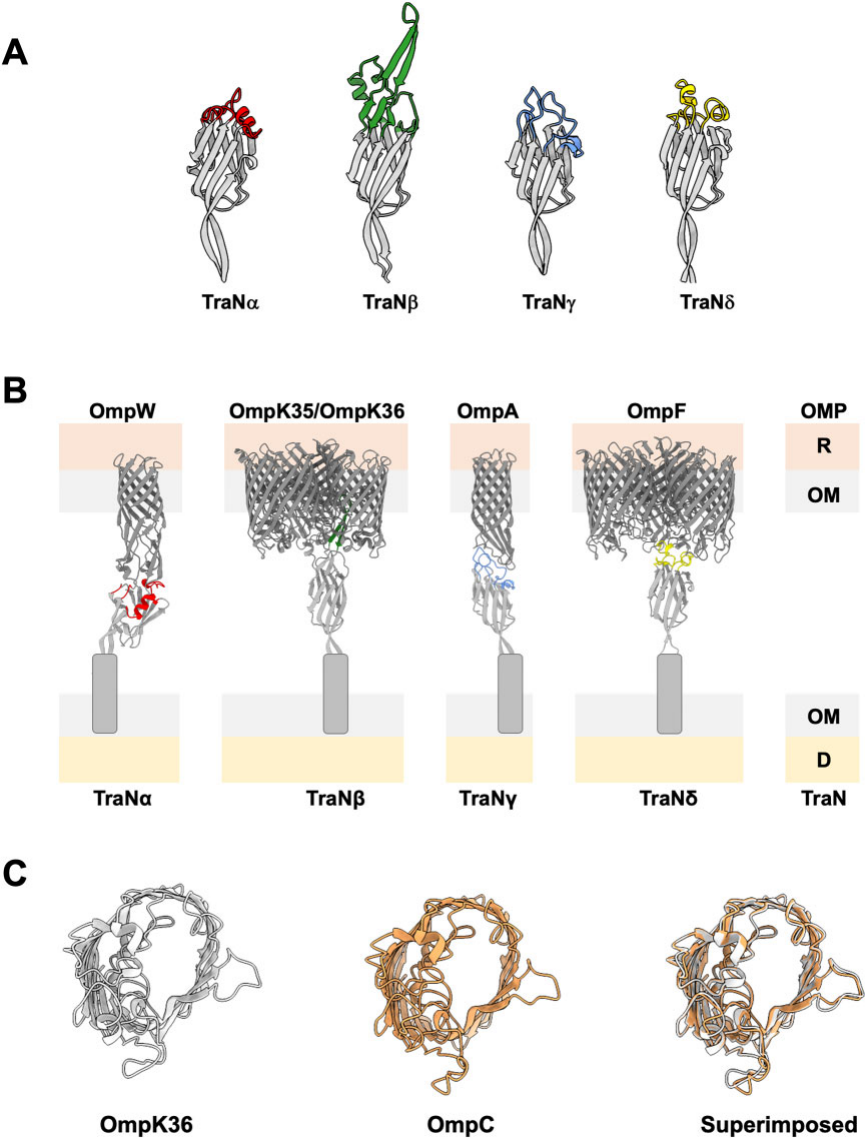
The TraN sensors. (**A**) The predicted structures of the evolved F-like plasmid-encoded TraNα, TraNβ, TraNγ and TraNδ tip sensors. The tip scaffold consists of conserved β-sheets, shown by gray ribbons. The colored structural motifs represent the surface exposed TraN sensor, each binding a specific receptor on the surface of the recipient. (**B**) The different TraN sensors in the donor (D), which recognize distinct OMPs in the recipient (R), mediate plasmid spread and conjugation species specificity. (**C**) The crystal structures of the *K. pneumoniae* OmpK36 (PDB ID: 6RD3) (59) and the *E. coli* OmpC (PDB ID: 2J1N) (63) are highly similar, yet the TraNβ sensor specifically recognizes recipients expressing OmpK36 but not OmpC.

The base consists of a conserved amphipathic alpha-helix that can potentially anchor the protein to the outer membrane (Figure 2B). Despite low sequence similarity, the tip folds into a conserved structure (Figure 2A), consisting mostly of β-sheets linked to a β-sandwich domain (Figure 2B). Structural differences between the different TraNs are mainly seen within the exposed loops of the tip domain, which functions as the TraN sensor (Figures 2B and 3A). The evolved TraN tip sensors fall into four dominant structural groups: TraNα (represented by R100-1 and pSLT), TraNβ (represented by pKpQIL and MV2), TraNγ (represented by F), and TraNδ (represented by MV1 and MV3) (57). Subtle differences in host selection have resulted in their classification into subgroups: TraNα of R100-1 and pSLT were classified as TraNα1 and TraNα2, respectively. Similarly, TraNβ of pKpQIL and MV2 and TraNδ of MV1 and MV3 were classified as TraNβ1 and TraNβ2 and TraNδ1 and TraNδ2, respectively (58).

The evolved TraN tip sensors selectively pair with specific OMPs in the recipient: TraNγ interacts with OmpA, TraNα interacts with OmpW, TraNδ interacts with OmpF and TraNβ interacts with both OmpK36 and OmpK35 (the *K. pneumoniae* OmpC and OmpF homologues respectively) (56), making it the only TraN variant currently known to cooperate with more than one OMP (Figure 3B). Structural determination of the TraNβ - OmpK36 complex and the predicted AlphaFold complex of TraNβ - OmpK35 revealed that the unique β-hairpin loop of the TraNβ sensor is inserted into one of the porin trimer subunits (56,58). This transcellular protein-protein interaction, which likely represents the molecular basis of MPS and conjugation specificity (56), parallels the recognition of bacterial hosts by the tail fibre RBPs of *Caudovirales* phages (10).

While low membrane abundance of OmpA (53) and OmpK35 (58) affect conjugation efficiency of F and pKpQIL plasmids respectively, lowering the abundance of OmpK36 does not affect conjugation of pKpQIL (58). Moreover, a single amino acid difference in loop 3 of OmpW between *E. coli* (N142) and *Citrobacter rodentium* (A142) affected their recipient activity. While *E. coli* OmpW was able to mediate MPS with both TraNα1 expressed by R100-1 and TraNα2 expressed by pSLT, OmpW of *C. rodentium* was only able to mediate MPS with TraNα2. An N142A substitution in OmpW of *C. rodentium* was sufficient to restore TraNα1-mediated MPS (58). This is consistent with what has been shown for OmpA, where a single amino acid mutation (G154D) inhibited TraNγ-mediated conjugation (51,52). Of note, Ried and Henning showed in 1987 that *E. coli* expressing the OmpA_G154D_ substitution was also resistant to specific phages (53), suggesting TraNγ and the phage RBPs share the same OmpA binding site. Mechanistically, the AlphaFold models suggest that the OmpW_N142A_ and OmpA_G154D_ substitutions cause steric clashes with TraNγ and TraNα1, respectively. Together, this shows that subtle differences in the recipient OMPs affect binding of TraN sensors and phage RBPs, suggesting that potential bacterial hosts can evolve to resist both phage infections and plasmid conjugation.

It is important to emphasise that while the TraN tip sensors have evolved multiple tertiary structures (Figure 3A), the structure of the major OMPs is highly conserved between species. Despite the close sequence and structural similarity of *K. pneumoniae* OmpK36 and *E. coli* OmpC (Figure 3C), TraNβ mediates MPS specifically with the former. This finding supports the hypothesis that conjugation is not a promiscuous mechanism of DNA transfer, but instead TraN functions as a sensitive sensor, enabling the selection of specific recipients. Conversely, OMPs are evolving in response to selective pressure, for example due to exposure to antibiotics. In *K. pneumoniae*, exposure to carbapenems have selected for truncation of OmpK35 and OmpK36 insertion mutants, both of which reduce antibiotic diffusion across the OM (59). The insertion mutants are characterised by single (D) or double (GD or TD) amino acids insertions into loop 3 of OmpK36, which constrict the porin pore (60). The OmpK36 loop 3 insertions not only synergise with the pKpQIL-encoded carbapenemase to increase carbapenem resistance, but also, inadvertently, reduce conjugation efficiency due to clashes with the β hairpin of the TraNβ tip and the porin (56,58). Interestingly, by and large clinical isolates expressing OmpK36 with loop 3 insertions already contain pKpQIL, suggesting that they may function on behalf of the plasmid as a proxy surface exclusion mechanism.

### TraN sensor influences the distribution of IncF plasmids in clinical isolates

**Figure 4.**
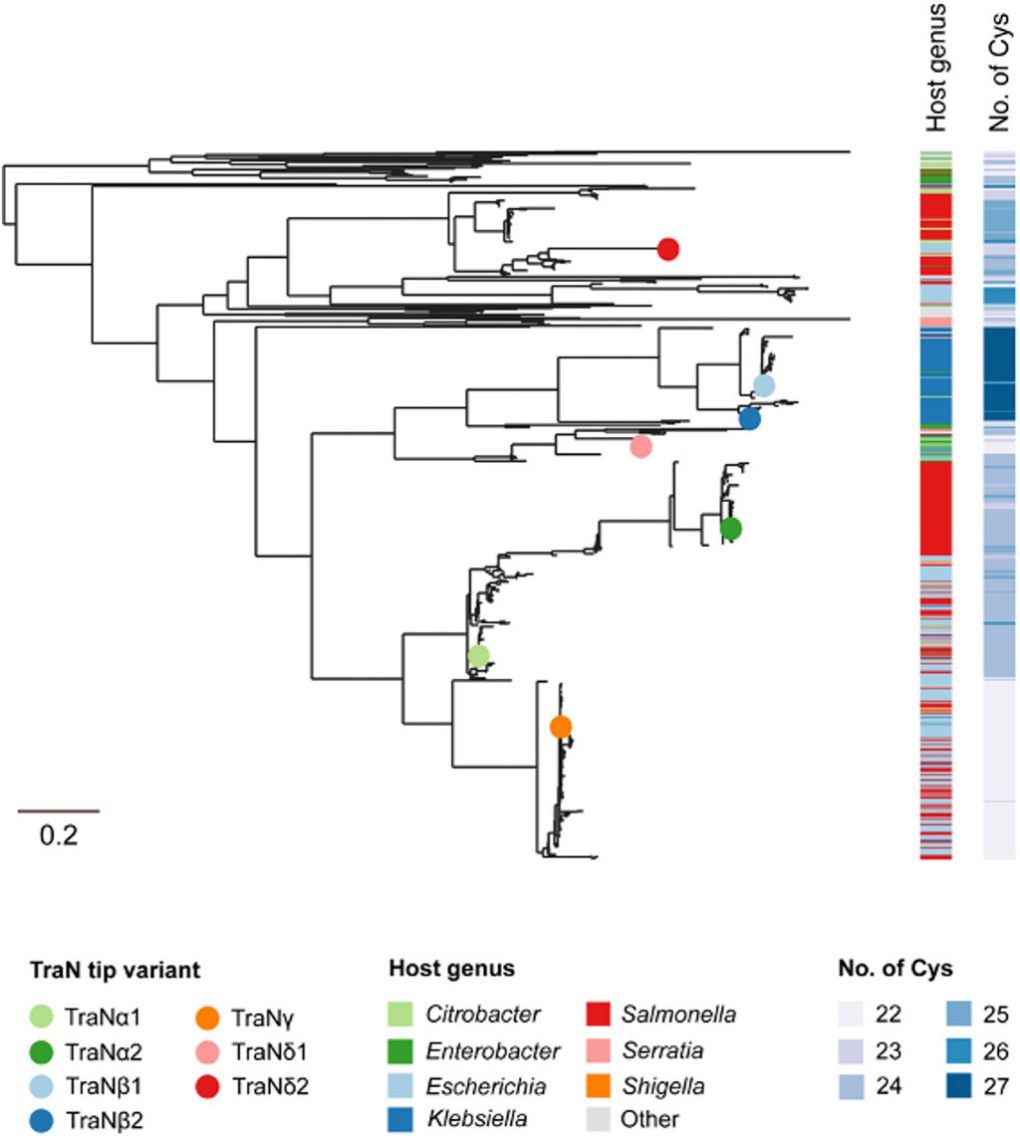
A phylogenetic tree of TraN. The tree (made with IQ-Tree) (64) consists of 639 TraN protein sequences (clustered following sequence alignment with Clustal Omega). These include 632 from Uniprot, filtered with 500–800 amino acids, ≤27 Cys residues and ≥ 30% amino acid similarity to TraN from the F plasmid. An additional seven TraN sequences from the R100-1 (TraNα1), pSLT (TraNα2), pKpQIL (TraNβ1), MV2 (TraNβ2), F (TraNγ), MV1 (TraNδ1) and MV3 (TraNδ2) reference plasmids were included and highlighted. Metadata blocks show the host genus of the plasmid and the number of Cys residues in TraN. The scale bar represents the number of substitutions per site. Each entry is associated with a UniProt accession code, and the structure prediction for each variant is available at: https://alphafold.ebi.ac.uk. An interactive version of the tree is available at: https://microreact.org/project/tran.

A TraN phylogenetic tree reveals clustering of the different tip variants into distinct clades, which are grouped with the Cys residue content and associated with one or more bacterial genera (Figure 4). Analysis of the different tip variants suggests that they could be divided into specialist (TraNβ and TraNγ) and generalist (TraNα and TraNδ) sensors, which exhibit narrow and broad host range, respectively (56). Importantly, while the specialist TraNs are predominantly found in a single species (e.g. TraNγ is found at a frequency of 92% in *E. coli*) they are also found at a lower frequency in other species (e.g. TraNγ is found at a frequency of 5.6% in *S*. enterica) (56). For illustration, the distribution of TraN sensors in a small selection of plasmids found in commensal and pathogenic Gram-negative bacteria is shown in Table 1. Taken together, these real-world distributions suggest that plasmids use TraN sensors to pick partners for dissemination within polymicrobial communities prior to conjugation.

**Table 1.** TraN in resistance and virulence plasmids

TraN	Protein ID	Plasmid	Resistance/Virulence	Origin strain
α	CDN85406.1	pEC958	Ciprofloxacin	ST131 Extraintestinal pathogenic *E. coli* (ExPEC)
β	ARQ19727.1	pKpQIL	Carbapenem	*Klebsiella pneumoniae*
β	WP_004152673.1	pKPN3	ESPBL^a^	*Klebsiella pneumoniae*
β	WP_015065533.1	pKDO1	ESPBL	*Klebsiella pneumoniae*
γ	QKQ01713.1	pSCU-103-1	MDR	Commensal *E. coli*
γ	QJT88315.1	pSCU-308-1	MDR	Commensal *E. coli*
α	QKN12826.1	pSCU-182-1	Ampicillin, Gentamicin	Commensal *E. coli*
δ	ANZ89826.1	pOZ172	MDR	*Citrobacter freundii*
α	AAL23498.1	pSLT	T3SS effectors	*Salmonella enterica* & *Salmonella Bongori*
α	WP_000821859.1	pUTI89	*cjrABC* genes	UTI89 Uropathogenic *E. coli* (UPEC)
γ	WP_000821827.1	pMAR7	BFP	Typical EPEC
γ	AMQ95459.1	pCERC4	Siderophores	*E. coli* ST58 (ExPEC)
δ	WP_001398575.1	pCss165	Heat stable enterotoxin	Enterotoxigenic *E. coli* (ETEC)

^a^Extended spectrum beta-lactamase

## CONCLUSIONS AND PERSPECTIVE

Most studies of F-like plasmid conjugation to date have been done using specific donor and recipient pairings (mainly *E. coli*) in either solid or liquid rich laboratory media. These investigations have shown that while MPS accelerates conjugation efficiency, promiscuous low-frequency transfer can happen even in its absence (doi: https://doi.org/10.1101/2023.06.21.545889; 56). However, F-like plasmid distribution in the real world suggests that where bacteria are experiencing shear forces, successful conjugation is reliant on MPS (56). This suggests that under physiological scenarios engagement of the pilus with a recipient is not sufficient for conjugation, but that via their TraN sensors plasmids pick their bacterial hosts and guide their own dissemination. Therefore, analogous to *Caudovirales* bacteriophages that use their tailed structures to bind bacterial surface receptors, it seems plasmids have also evolved a mechanism to selectively propagate in specific recipient species within polymicrobial communities. Interestingly, tail fibre RBPs and TraN isoform share similar OMP receptors. The questions of why some plasmids seem to avoid potential recipients (considering that that a copy of the plasmid remains in the donor), what are the evolutionary pressure that drive plasmid specialisation, and the reasons some potential recipients resist plasmid entry, are key questions for future studies.

The realisation that plasmids have evolved specific sensors to select their hosts represents a new viewpoint in plasmid biology, which could potentially be used to predict the spread of emerging resistance and virulence plasmids amongst pathogens.

## Data Availability

The data presented in this manuscript would be made available upon written request to the corresponding author.

## Notes

Present address: Wen Wen Low, Department of Biochemistry, Yong Loo Lin School of Medicine, National University of Singapore, Singapore.
