# Correction to ‘Plasmids pick a bacterial partner before committing to conjugation’

**DOI:** 10.1093/nar/gkad788

**Published:** 2023-09-23

*Nucleic Acids Research*, 2023; gkad678, https://doi.org/10.1093/nar/gkad678

In the originally published version of this manuscript, the Graphical Abstract contained an error. The correct version of the Graphical Abstract is include here:


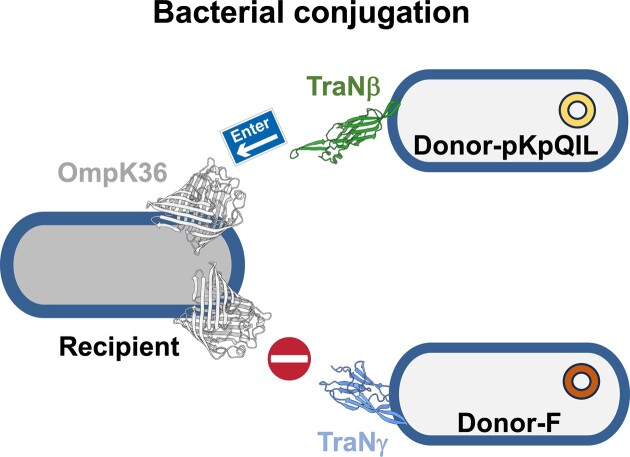


This error has been corrected online.

